# Advances in Tumor-Stroma Interactions: Emerging Role of Cytokine Network in Colorectal and Pancreatic Cancer

**DOI:** 10.1155/2019/5373580

**Published:** 2019-05-05

**Authors:** Chiara Bazzichetto, Fabiana Conciatori, Italia Falcone, Francesco Cognetti, Michele Milella, Ludovica Ciuffreda

**Affiliations:** ^1^Medical Oncology 1, IRCCS - Regina Elena National Cancer Institute, Rome 00144, Italy; ^2^Section of Oncology, Department of Medicine, University of Verona School of Medicine and Verona University Hospital Trust, Verona 37126, Italy; ^3^SAFU, Department of Research, Advanced Diagnostics, and Technological Innovation, IRCCS - Regina Elena National Cancer Institute, Rome 00144, Italy

## Abstract

Cytokines are a family of soluble factors (Growth Factors (GFs), chemokines, angiogenic factors, and interferons), which regulate a wide range of mechanisms in both physiological and pathological conditions, such as tumor cell growth and progression, angiogenesis, and metastasis. In recent years, the growing interest in developing new cancer targeted therapies has been accompanied by the effort to characterize Tumor Microenvironment (TME) and Tumor-Stroma Interactions (TSI). The connection between tumor and stroma is now well established and, in the last decade, evidence from genetic, pharmacological, and epidemiological data supported the importance of microenvironment in tumor progression. However, several of the mechanisms behind TSI and their implication in tumor progression remain still unclear and it is crucial to establish their potential in determining pharmacological response. Many studies have demonstrated that cytokines network can profoundly affect TME, thus displaying potential therapeutic efficacy in both preclinical and clinical models. The goal of this review is to give an overview of the most relevant cytokines involved in colorectal and pancreatic cancer progression and their implication in drug response.

## 1. Introduction

**Figure 1:**
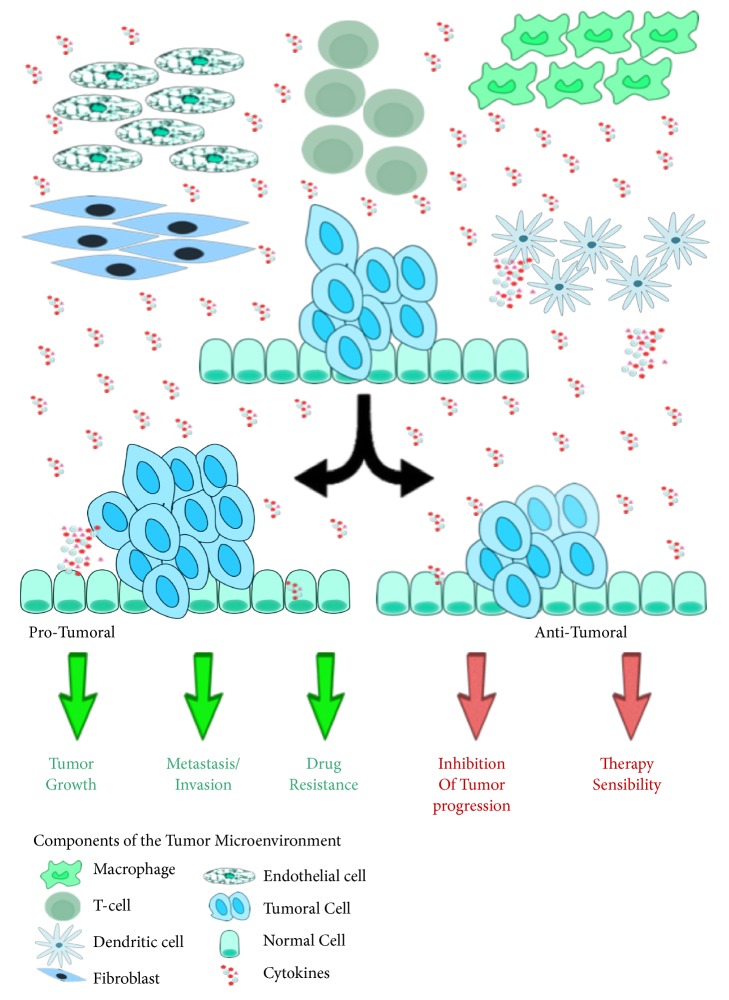
Schematic illustration of the cytokines role in tumorigenesis. Cytokines are released by both tumor and stromal cells, including immune cells like macrophages, B and T lymphocytes, dendritic cells, and endothelial cells and fibroblasts. The binding of cytokines to their receptors on surface of targeted cells causes the activation of intracellular signaling cascades with protumoral and/or antitumoral properties.

During the last years, it has been well recognized that cancer is not a single mass of transformed cells, but it is also composed by nonmalignant cells, such as Cancer-Associated Fibroblasts (CAFs), tumor infiltrating cells (T-cells, macrophages, and neutrophils), as well as vasculature with endothelial cells, soluble factors (cytokines and GFs), and the extracellular matrix, which are all together referred to as TME (Figure 1) [1, 2].

The connection between tumor and stroma is now well established and evidence from genetic, pharmacological, and epidemiological data supported the importance of microenvironment in tumor progression. However, several of the mechanisms behind TSIs and their implication in tumor progression remain unclear and need to be evaluated for their potential in pharmacological response.

The crosstalk between cancer cells and the surrounding TME may act through different processes, such as cell-to-cell direct contact, or by soluble factors. Indeed, one of the key players involved in intra- and intercellular communication is cytokines, like GFs and chemokines, which signal through both autocrine and paracrine fashion.

TSI represent one of most relevant contributors to the limited therapeutic success achieved by selectively targeting tumor cells. Indeed, not only does TME promote cancer invasion and metastasis, but it also provides resistance to chemotherapy, and cancer cells upregulate cytokines' expression proportionally to the progression of the disease. Understanding the mechanisms involved in TSI thoroughly in order to achieve “comprehensive” targeting of both cell autonomous progression mechanisms and TSI in advanced and metastatic colorectal and pancreatic tumor remains crucial [3, 4].

Herein, we will briefly describe current knowledge about the role played by chemokines and GFs in colorectal and pancreatic cancer and their treatment.

## 2. Cytokines Networks in Cancer

Cytokines are a set of soluble proteins and, through the binding to membrane receptors, they activate signal transduction pathways involved in several physiological and pathological mechanisms, thus providing complex networks of communication. Cytokines are released by both stromal and cancer cells in response to external stimuli; they can be clustered in families comprising GFs, chemokines, angiogenic factors, and interferons [5]. Various stroma cells can express cytokines, including immune cells, such as macrophages, B and T lymphocytes, dendritic cells, and fibroblasts and endothelial cells, thus affecting the behavior of cells around them (Figure 1).

Cytokines are redundant molecules, which regulate similar effects, due to their shared common receptors; moreover, they are pleiotropic, meaning that the cytokines-cytokines receptor interactions can, in turn, regulate a wide range of mechanisms, such as tumor cell growth and progression, angiogenesis, and metastasis [6]. However, several data demonstrated that cytokines can also display antitumoral properties, thereby highlighting a paradigm in cytokine role in affecting both pro- and antitumoral mechanisms (Figure 1) [7, 8]. Representative examples of the pleiotropic and controversial role of cytokines in TSIs are Interleukin-6 (IL-6) and Tumor Necrosis Factor-*α* (TNF)-*α*.

In multicellular organisms, IL-6 family plays an important role in communication and regulation of complex processes. Indeed, various cell types are involved in IL-6 secretion in response to different stimuli, such as immune reactions, response to infections, tissue injuries, hematopoiesis, and host defense. Due to the IL-6 involvement in homeostasis, it is not surprising that its uncontrolled signaling is associated with pathological processes like tumor initiation, progression, and metastasis [9, 10]. IL-6 family is composed by different cytokines (e.g., IL-6, IL-11, Oncostatin M (OSM) and Leukemia Inhibitory Factor (LIF)), which display the common ability to bind glycoprotein 130 (gp130) chain. This binding leads to the activation of the canonical IL-6-activated Janus Kinase- (JAK-) Signal Transducers and Activators of Transcription (STAT) pathway and Mitogen-Activated Protein Kinase (MAPK)/Extracellular Signal-Regulated Kinase (ERK) signaling, two of the most deregulated pathways involved in different stages of cancer development and progression [11]. The great complexity involved in IL-6 redundancy and pleiotropy is due to the various homo-/heterodimer receptor associations and subsequent intracellular signaling. gp130 plays a crucial role in signal transduction and its subunit is ubiquitously expressed; however, only IL-6 and IL-11 bind gp130 homodimer, while the other cytokines signal via heterodimers of gp130-LIF receptor or gp130-OSM receptor [12, 13].

TNF-*α* represents one of the most important activators of the NF-*κ*B “canonical pathway,” a master regulator of maximal cytokine expression; this mechanism in turn explains the cytokines autocrine loops with positive feedback [14]. Indeed, the binding of cytokines to their membrane receptors is able to activate transcription factors, which enhances cytokines gene transcription. TNF-*α* is a cytokine with a molecular weight of 26 kDa and it regulates different mechanisms (e.g., immunity, inflammation, cellular homeostasis, and tumor progression), according to its concentration [15, 16]. The cleavage of a membrane-bound protein (pro-TNF) from TNF-converting enzyme (ADAM17) allows the presence of a mature cytokine, which binds two main membrane receptors [17]. The binding to TNF Receptor-1 (TNFR1) (ubiquitously expressed) leads to the activation of the NF-*κ*B transcription factor, involved in the regulation of antiapoptotic genes (e.g., B-cell lymphoma-XL (Bcl-XL) and inhibitors of apoptosis) [7, 18]. On the other hand, due to its death domain, TNFR1 is able to bind caspase-8 and to activate the subsequent apoptotic pathway (through the activation of executor caspase-3 and -7) [7]. TNFR2 is mainly expressed by immune cells, but its role in cancer cells is less understood. The binding of TNF-*α* to TNFR2 in Colorectal Cancer (CRC) cell lines causes the activation of the phosphoinositide 3-kinase (PI3K)/AKT signaling through the phosphorylation of AKT, thus leading to cell proliferation. Furthermore, it has been shown that TNF-*α*-TNFR2 binding does not enhance MAPK/ERK signaling, as demonstrated by ERK inactivation [7, 19].

### 2.1. Chemokines

In the wide range of cytokines that affect TME, chemokines are one of the most interesting classes, due to their multiple roles played in TSI, which include TME's composition and function, tumor progression, and drug response [20].

Chemokines are chemotactic cytokines (8-10 kDa) secreted in several tissue environments and are involved in the regulation of inflammatory processes, in which they play a key role as chemoattractants [21]. To date, it has been demonstrated that there are about 50 types of chemokines and 20 seven-transmembrane-spanning G Protein-Coupled Receptors (GPCRs) in humans [22]. Ligand-receptor binding determines conformational changes, which allow the exposure of epitopes on the intracellular loops and carboxyterminal tail of the receptor; this in turn promotes the coupling with the functional heterotrimeric G proteins, which consist of *α*, *β*, and *γ* subunits. Ligand binding catalyzes the exchange of guanosine diphosphate for guanosine triphosphate on the G*α* subunit, which triggers the release of this subunit from the receptor and the G*βγ* subunits; consequently, signals are transmitted across the membrane and activate downstream effectors [23]. Chemokine binding to GPCRs also leads to the regulation of several both physiological and pathological processes. Indeed, during normal immune surveillance, chemokines induce cell polarization and migration of leukocyte, macrophages, and neutrophils in order to induce their homing in tissue injury or infection [24]. In pathological condition, such as cancer and inflammatory diseases, chemokines are able to activate specific signal transduction cascades, which are involved in proliferation, survival, and migration of cancer cells [24]. Besides the canonical chemokines receptors, 4 Atypical Chemokine Receptors (ACKRs) were recently identified: even if they display structural features similar to those of GPCR, they do not signal through the G proteins and they do not activate chemotaxis [25]. Indeed, ACKRs are involved in the regulation of the extracellular bioavailability of chemokines, their intracellular storage, and their cellular distribution in polarized cells [26].

Moreover, tumor cells not only are able to produce soluble chemokines but also overexpress chemokines receptors on their cell surface, thereby upregulating the autocrine mechanisms. In this way, signaling through chemokines is a complex mechanism of communication between tumor cells and TME through both paracrine and autocrine mechanisms [27].

Chemokines- (e.g., CXCL12 and IL-8-) GPCR binding mainly activates the MAPK/ERK signaling cascade and its downstream effectors involved in cell cycle progression and tumor cells proliferation, such as c-Myc and cyclin-D1 [28, 29]. GPCRs also activate the PI3K/AKT signaling pathway: once activated, AKT upregulates the oncoprotein Mouse double minute 2 homolog (Mdm2), the key antagonist of the p53 tumor suppresser gene, thereby promoting tumor cell survival [30].

One of the most relevant and characterized axes is CXC Ligand-12 (CXCL-12) (also known as Stromal Cell-Derived Factor (SDF)1-*α*)/CXC Receptor-4 (CXCR-4), crucially involved in homing and retention of Hematopoietic Stem Cells (HSC) in the bone marrow [31]. Consistently, food and drug administration approved plerixafor, a competitive inhibitor of CXCR4, for HSC transplantation: indeed, the blockade of CXCL12-CXC4 interaction results in mobilization of CD34^+^ hematopoietic stem cells to the peripheral blood by restoring bone marrow function [32, 33]. Moreover, it is been documented that CXCL12-CXCR4 axis is involved in a wide spectrum of cancer types, such as breast, colorectal, and pancreatic cancer [34–36]. CXCL12/CXCR4 is also responsible for apoptosis regulation via the activity of the Bcl-2 family members. For example, in acute myeloid leukemia cells, CXCL12-induced CXCR4 activation downregulates Bcl-XL expression, thereby shifting the balance from proapoptotic to antiapoptotic signaling [37]. Although CXCL12 does not contain the ELR (Glu-Leu-Arg) motif, it is one of the most angiogenesis-promoting chemokines [38]. Indeed, CXC chemokines are generally classified as promoter/suppressor of angiogenesis, according to the presence of ELR motif: ELR^+^ chemokines (CXCL1, CXCL6, and CXCL8) are positive regulators of angiogenesis; conversely, ELR^−^ chemokines (CXCL4, CXCL10, and CXCL14) display inhibitory features [39]. However, this classification does not always reflect the real activities performed by chemokines, as demonstrated by the role of CXCL12 in the regulation of vessels formation: indeed, it has been shown that CXCR4-defective mice display impaired vascular formation [40].

Another relevant chemokine axis involved in angiogenic mechanisms is the CXCL8- (also known as IL-8-) CXCR1/2 axis. IL-8 is a proinflammatory CXC ELR^+^ chemokine, identified for its role as “neutrophil chemotactic factor”: indeed, IL-8 mainly acts as a promoter of chemotaxis in target cells, primarily neutrophils but also other granulocytes, causing them to migrate toward the site of infection, where it promotes their chemotaxis and degranulation [41]. The biological effects of IL-8 are mediated through the binding of IL-8 to two cell-surface receptors, the GPCRs CXCR1 (IL-8RA) and CXCR2 (IL-8RB), which are present in various types of normal as well as tumor cells [42]. These receptors share 77% amino acid homology and retain common structural motifs, suggesting that these genes arose through gene duplication [43]. CXCR1 is activated by IL-8 and granulocyte chemotactic protein-2/CXCL6, whereas CXCR2 can be activated not only by IL-8 but also by many other CXC chemokines [43]. As a potent proangiogenic chemokine, IL-8 signaling induces Vascular Endothelial Growth Factor- (VEGF-) independent tumor angiogenesis: indeed, IL-8 stimulates endothelial cell migration and upregulates the two metallopeptidases MMP-2 and MMP-9 [44, 45]. Moreover, IL-8 is involved in a complex positive feedback loop with VEGF: Martin and colleagues have, indeed, demonstrated that IL-8 upregulates VEGF levels in endothelial cells, thereby activating VEGF receptors (VEGFR), through the transcription factor NF-*κ*B [46].

### 2.2. Growth Factors

In TME, cells can interact with each other also by the presence of polypeptide GFs, which act through the binding to specific cell-surface receptors, often with kinase activity. GFs are released in TME by different type of cells, such as tumor, endothelial, and mesenchymal cells; moreover, their cognate receptors can be expressed also by cells that are not those that released GFs, thereby representing a complex crosstalk between cells in TME. For example, mesenchymal cells predominantly release hepatocyte Growth Factor, which binds its receptor c-Met on the epithelial cells surface [47].

The general classification of GFs into 10 classes, according to targeted cells and functions, is summarized in Table 1 [48].

The interaction between the GFs and their receptors causes the activation of several intracellular signaling cascades, such as MAPK/ERK, PI3K/AKT, and JAK/STAT pathways, involved in supporting tumor progression and drug resistance. Indeed, GFs are involved in several hallmarks of cancer, such as uncontrolled proliferation, cellular motility, and angiogenesis, and they signal through both paracrine and autocrine mechanisms [49].

Transforming Growth Factor-*β* (TGF-*β*) is a multifunctional cytokine that regulates several physiological processes, such as cell development and differentiation, by acting as a negative regulator of tumor growth. Consistent with this role in cell proliferation, elements of this pathway (in particular the transcriptional factor Small Mother Against Decapentaplegic (SMAD)4) are commonly mutated in human cancers, thus promoting cell cycle progression, epithelial-mesenchymal transition, invasion, metastasis, and angiogenesis [50]. Once TGF-*β* binds its receptors, T*β*R-II recruits and phosphorylates T*β*R-I, which in turn phosphorylates its substrate complex SMAD2/3. This phosphorylation causes the dissociation of SMAD2/3 from the membrane, the association of a heterodimeric complex with SMAD4, and the translocation to the nucleus, where they act as transcriptional regulators of target genes, such as proapoptotic genes like BIM [51]. However, during the time, several studies showed that TGF-*β* also promotes protumoral effects, thereby displaying a controversial role in cancer. Indeed, it has been demonstrated that TGF-*β*/SMAD4 pathway interacts with the other canonical pathways involved in neoplastic transformation, such as MAPK/ERK and PI3K/AKT pathways, mainly due to phosphorylation events. For example, it has been demonstrated that TGF-*β*2 is able to activate ERK2 in breast cancer cell lines [52]. Conversely, ERK can phosphorylate SMAD2/3 in the linker region, thereby inhibiting their translocation to the nucleus [53].

Among GFs involved in cancer malignancy, Epidermal GF (EGF) promotes tumor growth and progression through the binding to erbB family receptors. erbB family comprises four transmembrane glycoproteins, with high molecular weight (170 to 185 kDa): EGF Receptor (EGFR) (HER1 or erbB1), erbB2 (HER2), erbB3 (HER3), and erbB4 (HER4). The EGF binding to the cell membrane receptor causes the dimerization of two EGFR monomers, which display tyrosine kinase activity on several substrates of both MAPK/ERK and PI3K/AKT pathways, in order to regulate biological processes including apoptosis and cellular proliferation [54]. According to this pivotal role in cell growth, mutations in EGFR gene, such as the copy number alteration, are frequently recurrent in cancer, thereby leading to EGFR overexpression and constitutive activation at the surface of tumor cells: for example, HER2 overexpression occurs in 15-30% of breast cancers and in 43-89% of the non-small cell lung carcinomas [55, 56].

Other prominent GFs involved in TSI are VEGF family, which comprise homodimeric soluble glycoproteins with a molecular weight of 45 kDa. VEGF is mainly involved in the angiogenic mechanism through its direct effect on endothelial cells, but over time its implication in promoting mitogenic stimuli in tumor cells has been also demonstrated, via autocrine self-regulation [57, 58]. VEGF-A is the most represented member of the VEGF family, which includes Placental Growth Factor (PLGF), VEGF-B, VEGF-C, and VEGF-D: VEGF-A binds to VEGFR1 and VEGFR2, VEGF-B and PLGF bind only to VEGFR1, and VEGF-C and VEGF-D bind to VEGFR2 [59]. Similar to EGFRs, the binding of VEGF to VEGFR induces the omo- or heterodimerization of receptors and the consequent activation of their kinase domain; the activity of VEGFR is regulated by the presence of two coreceptors: neuropilin-1 (NRP1) and NRP2. NRP1 and NRP2 exhibit 44% aminoacidic sequence identity and their expression is upregulated in cancer, supporting their role in oncogenic processes [58].

## 3. The Role of Cytokines in Colorectal and Pancreatic Cancer

### 3.1. Colorectal Cancer

CRC is the second main cause of worldwide cancer death, its pathogenesis is very complex, and it is influenced by multiple factors, associated with lifestyle (e.g., smoke, environmental factors, sedentary lifestyle, obesity, and/or hormones) or related to genetic predisposition (i.e., Chron's disease and/or colon polyps) [60]. Chronic inflammation represents one of the main causes involved in CRC progression and development [61]. A complex cytokines network characterizes CRC TME and, despite the role of inflammation in increasing CRC risk, different studies highlighted the correlation between immunity and a more desirable prognosis [62, 63].

TNF is one of the most characterized cytokines in CRC, probably due to the high presence of its receptors TNFR1 and TNFR2 in intestinal epithelial cells [19, 64]. TNF pathogenesis is associated not only with its levels but also with the specific receptor that it binds: low TNF levels are related to greatest percentage of cell migration but higher level of TNF with the inhibition of the physiologic wound closure, mediated by TNFR2 and TNFR1 binding, respectively [64, 65]. Stillie and her colleagues indeed demonstrated that, despite similar inflammation levels, mice lacking TNFR1 have reduced tumor and dysplasia incidence as compared to TNFR1 wild-type mice [64].

Moreover, even if during the time evidence has controversially highlighted TNF-*α* as both tumor promoter and suppressor, Grimm and colleagues demonstrated that TNF-*α* is involved in tumor growth, metastasis, invasion, and it is also correlated with positive lymph node stage and tumor recurrence in metastatic CRC (mCRC) patients [66, 67].

Despite the fact that TGF-*β* pathway is frequently altered in a high percentage of CRC patients, elevated levels of TGF-*β* have been observed in organoids derived from CRC patients. Indeed, TGF-*β* expression in TME is supported by the stromal cells compartment contribution (i.e., CAFs and endothelial cells), thus leading to enhancing the colonization capability of CRC cells at the initial phase of metastasis and consequent poor prognosis [68, 69]. Moreover, pharmacological inhibition of TGF-*β* signaling in the TME causes the reduction of metastases formation in* in vitro* patient-derived tumor organoids [69].

Elevated levels of IL-6 expression were observed in both serum and tissue of CRC patients [70, 71]. The production of IL-6 is mainly associated with NF-*κ*B activation and the involvement of IL-6 in CRC progression is actually accepted; indeed, a recent study demonstrated the direct correlation between IL-6 levels and Tumor Node Metastasis (TNM) stage and with less histological differentiation [72, 73]. Moreover, a recent meta-analysis confirmed the role of IL-6 levels with poor prognosis of both Overall Survival (OS) and disease-free survival of CRC patients, thus highlighting the role of IL-6 as an important biomarker in CRC diagnosis [74]. It has been further demonstrated that IL-6 is also involved in Microsatellite Instability (MSI), a mechanism observed in around 15% of CRCs [75]. Indeed, Tseng-Rogenski and her colleagues demonstrated the ability of IL-6 to induce MSI in* in vitro* CRC models, through the translocation of hMSH3 from the nucleus to the cytosol, thus blockading DNA mismatch repair [76].

Our group has recently demonstrated that the genetic background of CRC cell lines predicts specific chemokines patterns of expression. In particular, we showed that BRAF-mutation and PTEN-loss status are associated with higher levels of IL-8 production [77]. Indeed, IL-8 is another important cytokine involved in CRC and its levels are correlated with CRC progression and development of liver metastases [78]. Elevated serum levels of several cytokines, mainly released by tumor cells and CAFs, have a prognostic value and are also implicated in tumor aggressiveness and poor response to therapy: consistently, high levels of IL-8 in serum of patients correlate with a more advanced tumor stage [79]. Moreover, Lurje and collaborators demonstrated that germline polymorphisms of genes involved in tumor angiogenesis, such as IL-8 and VEGF, independently predict tumor recurrence in advanced status of CRC patients [79]. VEGF, indeed, represents the predominant angiogenic factor in CRC and preclinical experiments have correlated its expression with tumor progression, principally due to the angiogenesis and metastasis induction [80–82]. Furthermore, VEGF deletion, using somatic or siRNA knockout, leads to increasing of apoptosis and CRC sensitivity to chemotherapy [83, 84].* In vitro* results were also confirmed in CRC patients: VEGF expression is higher in tumor as compared to normal tissue and elevated levels in tissues are associated with an advanced stage of the disease [85, 86].

### 3.2. Pancreatic Cancer

Pancreatic cancer is one of the most aggressive tumors characterized by a very poor prognosis and by the refractoriness to conventional therapies [87]. Despite the absence of a strong prognostic factor, different studies are focused on the analysis and identification of the putative role of proinflammatory and angiogenic factors in pancreatic cancer patients. Indeed, both pro- and anti-inflammatory cytokines/chemokines are overexpressed in pancreatic cancer [88]. Ebrahimi and his colleagues demonstrated that serum of pancreatic carcinoma patients displays higher levels of IL‐6, IL‐10, IL‐8, and IL‐1RA as compared to serum of healthy patients [4]. In particular, IL-6 levels correlate with weight loss and with a worse prognosis [4, 89]. The importance of IL-6 in pancreatic cancer is due to its release not only by cancer cells but also by stromal cells, thus leading to the progression of pancreatic intraepithelial neoplasia and the development of Pancreatic Ductal Adenocarcinoma (PDAC) [90]. These results also support the relevance of the constitutive activation of STAT3 pathway in affecting a malignant phenotype of pancreatic cancer [91].

In* in vitro* and* in vivo* pancreatic cancer samples, IL-8 overexpression is associated with the increasing production of VEGF and metastatic progression in hypoxic condition, through the MAPK/ERK pathway activation [92]. The IL-8-mediated invasive and migration capability is allowed by cooperation with both SDF1-*α* in TME and MMP-2 activity [93, 94]. The correlation between IL-8 and clinicopathological status of pancreatic cancer patients is also confirmed by the CXCR1 upregulation in tissue derived from patients, which are characterized by poor prognosis [95].

NF-*κ*B pathway is one of the most activated signaling pathways in PDAC cells and patient-derived tissues and its activation is principally due to TNF-*α*. Consistent with this evidence, TNF-*α* levels are high in patients affected by pancreatic cancer and correlate with advanced status of the neoplasia [96, 97]. Moreover, TNF-*α* affects tumor cell growth and invasion in pancreatic tumor both* in vitro* and* in vivo* [98]. Ringel and colleagues also identified the aberrant expression of ADAM17, the TNF-*α* processing enzyme, and its role in invasion of both PDAC cell lines and tissue derived from patients [99].

The controversial role of TNF described in CRC is also observed in PDAC: albeit the TNF exposure of tumor-bearing mice increases tumor growth, TNF plays also antitumorigenic function through TNFR1. Indeed, the presence of TNFR1 is necessary to ensure better immunosurveillance, mediated by increased infiltration of CD8^+^ T cells [100].

Another mutation involved in pancreatic tumor progression is undoubtedly associated with SMAD4 gene. This tumor suppressor is inactivated in around 55% of PDAC with the homozygous deletion of both alleles or with the loss of one and the mutation in the other one [101]. SMAD4 is the mediator of TGF-*β* signaling and its association with tumor growth and metastasis in PDAC is currently known [102, 103]. A recent study from Zhao and colleagues showed the potential prognostic role of TGF-*β*: indeed, higher serum levels of TGF-*β* were detected in PDAC patients as compared to healthy patients or to benign pancreatic conditions; levels of TGF-*β* also identified pancreatic cancer stage (I-II versus III-IV) and correlated to the reduction of survival and poor prognosis [104].

A clinical significance to growth-regulated oncogene-*α* has been assigned by Lian and collaborators. In a recent study, they observed higher level of this chemokine in pancreatic cancer tissues as compared to normal ones, and the expression was correlated with TNM stage and metastases localization, thus leading to significant poor survival of patients [105].

Despite several evidences on the association of specific cytokines/chemokines and the modulation of pancreatic cancer patient survival, a recent study highlighted the importance of the general inflammatory status definition to develop a better target combination strategy. Indeed, this large prospective clinic-based study showed how combined marker of inflammation coincides with greater mortality in pancreatic cancer patients [106].

## 4. Involvement of Cytokines Patterns in Cancer Therapeutic Choice

As mentioned above, TME and TSI also increase drug-resistance development of cancer cells, thus leading to the need of better understanding of the mechanisms behind acquired tumor resistance, which remain crucial to determine overall patient benefit [107].

Mutational status in CRC is a strong predictor for OS, not only in the metastatic setting but also in earlier stages, and it is involved in drug resistance development [108]. Furthermore, mutations are often used as a biomarker to select patients who would benefit from a specific therapeutic approach: indeed, in patients with mCRC, OS has improved mainly due to the use of targeted therapies, but survival improvement is linked to proper selection of patients who could benefit from these treatments. For example, only patients lacking mutations in KRAS or NRAS benefit from EGFR monoclonal antibodies (cetuximab and panitumumab) treatment [108]. Indeed, panitumumab is currently used in combination with chemotherapy in first and second line and as a monotherapy in chemorefractory KRAS-wild-type CRC patients [109].

Another biologic therapy targeting angiogenesis in mCRC is represented by bevacizumab, a humanized recombinant monoclonal antibody directed against VEGF-A. Bevacizumab is recommended as first- and second-line treatment in combination with chemotherapy, for KRAS-mutated stage IV mCRC patients. However, several studies showed that the clinical benefit from anti-VEGF therapy appears to be independent of KRAS status and predictive biomarkers of sensitivity/resistance have not been yet identified [110]. A recent study demonstrated that IL-8 polymorphisms (c.-251T>A) correlate with a worse Progression-Free Survival (PFS) in KRAS-mutated bevacizumab-treated mCRC patients, consistent with the role of IL-8 in angiogenesis and thus representing an escape mechanism from VEGF-targeted treatment [111].

During the last years, an increasing number of evidences have highlighted the role of IL-8 as a putative prognostic/predictive biomarker in CRC. For example, Lurje and colleagues demonstrated that germline polymorphisms of IL-8 (T2251A) and VEGF (C+936T) are associated with a higher risk of developing tumor recurrence in stage III CRC patients [79]. Furthermore, Rubie and her colleagues showed that IL-8 levels have a prognostic value and are also implicated in tumor aggressiveness and poor response to therapy: indeed, they demonstrated that IL-8 production is associated with CRC progression, including liver metastases development [78]. A significant number of* in vitro* and* in vivo* preclinical studies support the importance of IL-8-CXCR1/2 signaling in promoting tumor progression and multiple small-molecule antagonists and humanized monoclonal antibodies are under investigations [112]. Based on this evidence, IL-8 and its receptors CXCR1/2 could represent a novel therapeutic target in CRC to sensitize cancer cells toward chemotherapy [113]. Indeed, treatment with an inhibitor of CXCR2, SCH-527123, alone and in combination with oxaliplatin, is effective in synergistically inhibiting proliferation and angiogenesis and enhancing chemosensitivity in CRC cells and xenografts [113].

Matsusaka and colleagues investigated also the correlation between IL-6 (rs2069837, rs1800795) and STAT3 (rs744166, rs4796793) polymorphisms and the outcomes in a phase III mCRC trial of first-line bevacizumab-based chemotherapy, thus demonstrating that IL-6 genotype may be a useful predictive and prognostic biomarker in mCRC patients [114]. Even if IL-6/STAT3 signaling is involved in CRC progression, clinical trials that target IL-6 pathway are currently missing. After the failure of anti-IL-6 antibodies and the controversial results of chimeric murine-human monoclonal anti-IL-6 antibody siltuximab, the anti-IL-6R antibody tocilizumab and the small JAK1 and 2 inhibitor ruxolitinib were developed, but no clinical trial has been developed for cancer treatment [115].

TME and stroma are the most therapeutic barriers in drug response of pancreatic cancer by affecting treatment responses and PDAC patients survival [116]. IL-6/JAK/STAT axis represents a key pathway involved in PDAC progression. Indeed, Xing and collaborators recently demonstrated that IL-6 silencing causes increasing of apoptosis, thus reducing tumorigenicity of cancer cells. Moreover, IL-6 downregulation by gene-silencing enhances the sensitivity of pancreatic cells to gemcitabine [117].

Due to the described evidence of TNF-*α* implication in pancreatic cancer progression, Egberts and his group investigated the effects of the chimeric monoclonal antibodies infliximab and etanercept on PDAC cells in both* in vitro* and* in vivo* models. Although no significant effects on cell proliferation and invasiveness were observed* in vitro*, strong effects on reducing number of liver metastases were detected in orthotopic xenotransplantation mice models [98]. However, even if TNF-*α* seems to be relevant in PDAC patients, a phase I/II study for the combination of chemotherapy (gemcitabine) and TNF-*α*-inhibitor (etanercept) failed to demonstrate a synergism in PDAC patients [118].

A recent phase II clinical trial with the combination of gemcitabine and galunisertib, a TGF-*β* inhibitor, showed synergistic effects of the two drugs, as demonstrated by an improvement of OS and PFS in stage II to stage IV unresectable PDAC patients [119].

## 5. Conclusions

Even though it is now well established that TME, with inflammation and inflammatory mediators (such as chemokines, GFs, and angiogenic factors), plays an important role in promoting tumor progression, metastasis, and drug resistance, many of the mechanisms underlying TSIs are to be identified. Indeed, interactions among cancer cells and between cancer cells and the surrounding microenvironment can affect the sensitivity of tumor cells to targeted therapy/chemotherapy. Understanding the role of cytokines in TSIs could be crucial to predict pharmacological responses to specific antagonists and to build the rationale for novel therapeutic combinations in cancer treatment.

## Figures and Tables

**Table 1:** GF classification.

GFs family	Abbreviation	Receptors	Target cells	Role in cancer
Platelet Derived Growth Factor	PDGF	PDGFR*α*/*β*	Epithelial and endothelial	Blood vessel formation, cell migration and metastasis

Vascular Endothelial Growth Factor	VEGF	VEGFR1/2/3 and coreceptors Neuropilin1/2	Endothelial and tumor	Mitogenic stimuli

Epidermal Growth Factor	EGF	EGFR	Mesenchymal, epithelial and tumor	Mitogenic stimuli and metabolism increasing

Fibroblast Growth Factor	FGF	FGFR1-4	Epithelial and mesenchymal	Mitogenic and angiogenic stimuli

Transforming Growth Factor-*β*	TGF	TGF-*β* type I (T*β*R-I), type II (T*β*R-II) and type III (T*β*R-III)	Tumor	Tumor suppressor

Insulin-like Growth Factor	IGF	IGF-IR and IGF-IIR	Tumor	Upregulation of metabolism, growth and survival

Hepatocyte Growth Factor	HGF	c-Met	Epithelial	Mitogenic stimuli and angiogenesis

Neurotrophin	n.a.	Tropomyosin receptor kinase (Trk) A/B/C and neurotrophin receptor P75	Tumor	Cell survival (Trk) and death (P75)

Ephrin	Eph	EphrinA/B	Tumor	Tumor suppressor/promoter

Angiopoietins	ANG	TIE-2 tyrosine kinase receptor	Endothelial and tumor	Angiogenesis
